# Routine chemoprophylaxis for deep vein thrombosis in Indian patients: Is it really justified?

**DOI:** 10.4103/0019-5413.40265

**Published:** 2008

**Authors:** Lalit Maini, Hemant Sharma

**Affiliations:** Department of Orthopaedics, Maulana Azad Medical College, New Delhi, India; 1 Department of Orthopaedics, Aruna Asaf Ali Govt. Hospital, Rajpur Road, New Delhi, India

Sir,

We read with interest the article “Routine chemoprophylaxis for deep vein thrombosis in Indian patients: is it really justified?”. We differ with the observations made by the authors.^1^

Firstly, the incidence mentioned by the authors i.e. 7.2% is significant according to us and requires more than just close monitoring.

Secondly, there are quite a few studies in the Indian literature contrary to the authors' observation. They might not be accessible on Pubmed and hence were not mentioned by the authors. We could find out quite a few studies in the Indian literature/on Indian patients with an incidence of deep vein thrombosis (DVT) higher than that reported in this study. Shead,^2^ Nagi,^3^ Sharma,^4^ Rajgopalan N,^5^ Bhan,^6^ Maini^7^ reported incidences of 28%, 8%, 19.6%, 23.3%, 7.8%, 9.9% respectively. Various published studies from Agarwala^8^–^10^ showed incidences of 54%, 34.4% and 43.2% with prophylaxis and 60% without prophylaxis. Rajgopalan, Agarwala and Maini used low molecular weight heparin for prophylaxis. Shead used 125 fibrinogen uptake to determine the incidence of deep vein thrombosis. Shead, Nagi and Sharma used no prophylaxis and Bhan's is the subgroup which received no prophylaxis.

Here we would also like to draw attention to certain points in the study published which could have affected the final outcome. The average age in this study was though 60 years for males (range 25-90 years) and 62 years for females (29-94 years), but the range is very wide. Age has beyond doubt proved to be a significant factor in the development of deep vein thrombosis. The incidence of venous thromboembolism increases exponentially with age. Age is additive to other factors for thromboembolism. Age is also a risk factor independent of other risk factors.^11^ The inclusion of young patients less than 40 years of age might also have been one of the reasons for low incidence of deep vein thrombosis in this study.

Another reason according to us is the miscellaneous surgeries (29, i.e. 23% of the total surgeries). The interlocking nail femur/tibia constituted 11 (8.8%) and other surgeries were 18 (14.4%). We are not sure how many of these were acetabular surgeries, (one patient who underwent acetabular reconstruction developed DVT) and what percentage of surgeries involved was ankle and knee trauma surgeries. Joint replacement and hip fracture surgery definitely have a higher incidence of DVT compared to other lower limb trauma surgeries.^11^

Distal deep vein thrombosis may look benign as mentioned by the authors. The contradictory evidence from various studies has suggested that isolated calf vein thrombi may propagate rapidly and fatal pulmonary embolism from isolated calf vein DVT is a significant risk.^10^
